# Mongolian warm acupuncture improves colonic barrier function and immune responses via the TLR4/MyD88/NF-κB pathway in insomnia rats

**DOI:** 10.1016/j.ibneur.2026.07.014

**Published:** 2026-07-25

**Authors:** Gaowa, Zhiying Bao, Zengqiang Wang, A. Gula, Hong Yu

**Affiliations:** a Mongolian Medicine School, Inner Mongolia Medical University, No. 5 Xinhua Street, Hohhot, China; b School of Nursing, Baotou Medical College, Inner Mongolia Autonomous Region, Baotou 014040, China; c Baotou Medical College Institute of Chronic Disease Nursing, Inner Mongolia Autonomous Region, Baotou 014040, China; d College of Traditional Chinese Medicine, Baotou Medical College, Inner Mongolia Autonomous Region, Baotou 014040, China; e Mongolian and Traditional Chinese Medicine Hospital, Inner Mongolia Autonomous Region, Baotou 014040, China

**Keywords:** Insomnia, Mongolian warm acupuncture, Colonic Barrier, Immune Responses, TLR4

## Abstract

Insomnia is a prevalent sleep disorder associated with intestinal barrier dysfunction and immune dysregulation. Mongolian warm acupuncture (MWA) has shown therapeutic potential in insomnia; however, its effects on intestinal permeability and underlying mechanisms remain unclear. In this study, an insomnia rat model was established using chronic unpredictable mild stress combined with para-chlorophenylalanine (PCPA). To evaluate the therapeutic effects of MWA, comparative analysis was conducted among different groups, including Control, Model, MWA, Estazolam, and sham MWA groups. MWA group showed significantly improvement on sleep quality, cognitive performance, and anxiety-like behavior and alleviated colonic tissue damage. MWA treatment attenuated intestinal barrier dysfunction by modulating the expression of ZO-1, Occludin, and Claudin-4. In addition, MWA reduced inflammatory responses by decreasing TNF-α, IL-1β, and IL-6 levels while increasing IL-22 expression. Furthermore, MWA suppressed activation of the TLR4/MyD88/NF-κB signaling pathway. These findings suggest that MWA exerts protective effects against insomnia-associated intestinal barrier dysfunction and inflammation and may represent a promising non-pharmacological therapeutic strategy for insomnia.

## 1 Introduction

Insomnia is a common sleep disorder characterized by persistent difficulty initiating or maintaining sleep, leading to impaired sleep quality and reduced sleep duration, often accompanied by daytime dysfunction (Ohayon, 2002). It affects approximately 10–30% of the global population and is increasingly recognized as a systemic disorder associated with neuroimmune and metabolic dysregulation (Riemann et al., 2023, Cao et al., 2017). Chronic sleep deficiency is linked to increased risks of psychiatric, cardiovascular, and metabolic diseases (Irwin, 2019). Currently, pharmacological therapy remains the primary clinical treatment for insomnia, with benzodiazepines and non-benzodiazepine hypnotics being the most commonly prescribed medications (Chavez-Mendoza et al., 2025). Although these agents demonstrate clinical efficacy, their long-term use is associated with adverse effects, including tolerance, dependence, cognitive impairment, and increased risk of falls (Watson et al., 2023, Chapoutot et al., 2021). Therefore, identifying safe and effective therapeutic strategies for insomnia remains an important clinical priority.

Previous studies indicate that intestinal barrier dysfunction is closely associated with sleep disorders (Andersen et al., 2023, Gao et al., 2019, Li et al., 2021). Increased intestinal permeability can promote translocation of microbial-derived molecules and endotoxins into systemic circulation, triggering systemic and neuroinflammatory responses that disrupt sleep regulation (Cheng et al., 2025, Gao et al., 2020). Barrier disruption can induce pro-inflammatory cytokine release (Veler, 2023), including interleukins and tumor necrosis factor-α (Yu et al., 2020, Wu et al., 2023, Yang et al., 2023), partly via Toll-like receptor 4 (TLR4)-mediated signaling (Hergenhan et al., 2020). Activation of TLR4 subsequently triggers MyD88-dependent pathways and nuclear factor kappa B (NF-κB), promoting sustained inflammatory responses and chronic low-grade systemic inflammation (Zhao et al., 2021). Experimental studies further demonstrate that sleep deprivation disrupts intestinal barrier integrity and increases intestinal permeability (Irwin, 2015), highlighting the bidirectional interaction between sleep regulation and intestinal barrier function.

Mongolian warm acupuncture (MWA) is a traditional therapy in Mongolian medicine, that integrates mechanical and thermal stimulation at specific acupoints to regulate physiological function and systemic homeostasis (Shao et al., 2022). It has been widely applied in the treatment of musculoskeletal, insomnia and nervous diseases (Si et al., 2024, Bo et al., 2016, Cao et al., 2024). Emerging evidence suggests that MWA can modulate microRNA expression profiles and normalize serum metabolic disturbances in insomnia models (Bo et al., 2016, Bo et al., 2017). In addition, MWA has been shown to influence the gut microbiota composition and host metabolic profiles in insomnia models, ncluding improving microbial diversity, reducing the abundance of Romboutsia, and increasing Lactobacillus and Clostridium sensu stricto in insomnia rats (A et al., 2019, Yu et al., 2022, Wu et al., 2025). However, the role of Mongolian warm acupuncture in regulating intestinal permeability and immune function remains unclear.

In this study, an insomnia rat model was established using chronic unpredictable mild stress combined with para-chlorophenylalanine (PCPA). Rats were divided into Control, Model, MWA, Estazolam, and sham MWWA (SMWA) groups to evaluate the therapeutic effects of MWA. Behavioral assessments, along with histopathological and molecular analyses, were performed to assess sleep behavior, colonic morphology, inflammatory cytokines, tight junction proteins, and TLR4/MyD88/NF-κB signaling. This study aimed to elucidate the protective effects and underlying molecular mechanisms of MWA in insomnia-associated intestinal barrier dysfunction and immune dysregulation, with a particular focus on the TLR4/MyD88/NF-κB signaling pathway.

## 2 Methods and materials

### 2.1 Animals and housing conditions

A total of 50 male Sprague Dawley (SD) rats, weighing from 180 to 220 g, were obtained from SPF (Beijing) Biotechnology Co., Ltd. (Beijing, China; Animal Production License No. SCXK (Beijing) 2019–0010). All experiments were approved by the Ethics Committee of Baotou Medical College, Inner Mongolia University of Science and Technology.

Rats were housed in a controlled animal facility under standard laboratory conditions, with an ambient temperature of 20–26 °C and relative humidity of 40–60%. All rats were acclimated to the laboratory environment for one week before the experiment.

### 2.2 Establishment of insomnia model

After acclimation, rats were randomly assigned into five groups (n = 10 per group): Control, Model, MWA treatment, Estazolam treatment, and sham MWA treatment (SMWA) groups. Except for the Control group, all rats underwent insomnia model induction using a combination of chronic unpredictable mild stress (CUMS) and para-chlorophenylalanine (PCPA) administration.

To simulate chronic psychological stress conditions associated with human insomnia, rats were subjected to CUMS for 14 consecutive days. During this period, rats were randomly exposed to one stressor per day, including tail clamping (1 min), cold-water swimming at 4 °C (5 min), food deprivation (24 h), water deprivation (24 h), circadian rhythm reversal (24 h), wet bedding exposure (24 h), or cage tilting at 45° (24 h). Stressors were applied in a randomized sequence to prevent adaptation. Following the CUMS exposure, insomnia was further induced by intraperitoneal injection of para-chlorophenylalanine (PCPA, 400 mg/kg) once daily for two consecutive days. The Control group received no stress or PCPA treatment, whereas the SMWA, estazolam, and MWA groups underwent the same modeling procedures as the Model group. Then, behavioral tests were performed to assess insomnia-related behavioral alterations and cognitive function.

### 2.3 Group and MWA treatments

As the insomnia model was established, rats received corresponding interventions for seven days. The Control group received no treatment. Rats in the Model group were handled and restrained similarly to the treatment groups but without intervention. Rats in the Estazolam group received estazolam (0.2 mg/kg) via oral gavage once daily. Rats in the MWA group received active warm acupuncture treatment. Rats in the SMWA group received sham treatment using blunt needles placed in contact with the skin surface without penetration, followed by identical thermal stimulation.

**Fig. 1:**
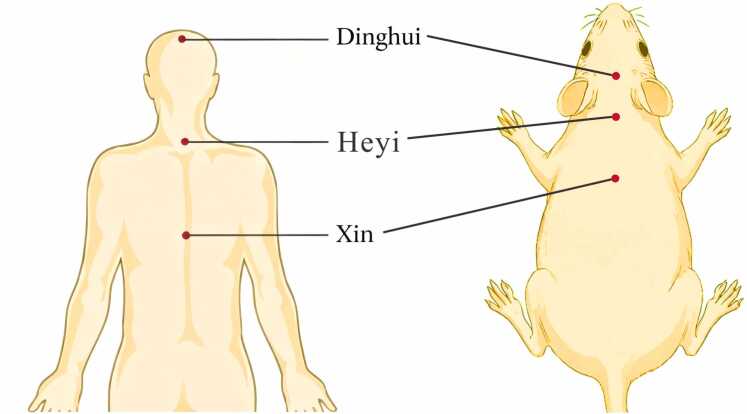
Schematic illustration of acupoint locations used for Mongolian warm acupuncture treatment. The three acupoints used in this study, including Dinghui, Heyi, and Xin, are shown on both the human body reference and the rat model.

For Mongolian warm acupuncture treatment, three acupoints were selected based on Traditional Mongolian Therapeutics and Experimental Acupuncture Techniques (2012 edition): “Dinghui” point (located at the intersection of the midline extending vertically from the anterior hairline and the horizontal line connecting the superior margins of both auricles), “Heyi” point (located at the midpoint of the depression over the first thoracic vertebra), and “Xin” point (located at the midpoint of the depression over the seventh cervical vertebra) (Fig. 1). During treatment, rats were gently restrained using custom fixation bags. Sterile Mongolian silver acupuncture needles (length 25 mm, diameter 0.5 mm) were inserted obliquely into the selected acupoints at a depth of approximately 0.3–0.5 cm. Needles were connected to an MLZ-III Mongolian intelligent acupuncture therapy device (developed by the School of Mongolian Medicine, Inner Mongolia Medical University; Patent No. ZL201120058078.0), which provided controlled thermal stimulation. The treatment temperature was maintained at 40 °C, and needles were retained for 15 min per session. Skin at stimulation sites was monitored to avoid thermal injury.

### 2.4 General physiological observations

Rats were monitored daily throughout the experimental period for general physiological conditions, including food intake, body weight, fecal appearance, fur condition, and spontaneous activity. Rat weight was recorded before model induction, after model establishment, and at the end of the treatment period.

### 2.5 Pentobarbital sodium-induced sleep test

After 7 days of treatment, rats were intraperitoneally injected with pentobarbital sodium (45 mg/kg, 1% solution), and were continuously observed after administration. Sleep onset was defined as loss of the righting reflex for at least 1 min, and awakening was defined as recovery of the righting reflex. Sleep duration was calculated as the interval between loss and recovery of the righting reflex.

### 2.6 Morris water maze test

Spatial learning and memory were assessed using the Morris water maze test. Behavioral assessments were performed at three time points: after the first PCPA injection, prior to treatment initiation, and 60 min after the final treatment. The water temperature was maintained at 25 ± 2 °C, and the hidden platform was submerged approximately 1 cm below the water surface. The test consisted of an acquisition training phase followed by a probe trial. During acquisition training, rats were placed into the pool facing the wall and allowed to search for the hidden platform for up to 120 s. If a rat failed to locate the platform within 120 s, it was gently guided to the platform and allowed to remain there for 15 s. Training sessions were conducted twice daily at 12 h intervals for three consecutive days. Escape latency and swimming distance were automatically recorded. On day 4, a probe trial was performed after removal of the platform. The number of platform crossings and time spent in the target quadrant were recorded to evaluate spatial memory.

### 2.7 Open field test

Spontaneous locomotor activity and anxiety-like behavior were assessed using the open field test (OFT) with an automated behavioral tracking system (OFT−100). Behavioral assessments were performed at three time points: after the first PCPA injection, before treatment initiation, and 60 min after the final treatment session. Rats were allowed to acclimate to the open field arena for 3 min, after which each rat was placed in the center of the arena and recorded for 5 min using an overhead camera system. The arena was cleaned with 75% ethanol between trials to eliminate residual odors. Behavioral parameters, including rearing frequency, total movement distance, and immobility time, were automatically recorded and analyzed using the behavioral analysis software.

### 2.8 Hematoxylin and eosin staining

Colonic tissues were fixed in 4% paraformaldehyde, processed routinely, and embedded in paraffin. Tissue sections (5 μm) were prepared, followed by deparaffinization, rehydration, hematoxylin and eosin (H&E) staining, and mounting with neutral resin. Histopathological changes, including villus morphology and crypt structure, were examined under a light microscope. Mucosal thickness and crypt depth were quantified using ImageJ software.

### 2.9 Enzyme-linked immunosorbent assay (ELISA)

Six rats were randomly selected from each group. Colonic tissues were homogenized and centrifuged at 12,000 rpm at 4 °C, and the supernatants were collected for analysis. The concentrations of tumor necrosis factor-α (TNF-α), interleukin−1β (IL-1β), interleukin−6 (IL-6), and interleukin−22 (IL-22) were measured using commercial ELISA kits according to the manufacturers’ instructions.

### 2.10 Western blot analysis

Total proteins were extracted from homogenized colon tissues. Protein concentration was determined using a BCA protein assay kit. Protein samples were denatured, separated by SDS-PAGE, and transferred onto PVDF membranes. The membranes were blocked with 5% non-fat milk at room temperature for 1.5 h and washed three times with TBST, followed by overnight incubation at 4 °C with primary antibodies against Occludin (1:1000), TLR4 (1:1000), NF-κB p65 (1:5000), ZO−1 (1:5000), MyD88 (1:5000), α-actin, and β-actin. For tight junction proteins, including ZO−1, Occludin, and Claudin−4, α-actin was used as the loading control. For TLR4, MyD88, and NF-κB p65, β-actin was used as the loading control. After washing, membranes were incubated with appropriate secondary antibodies at room temperature for 2 h. Protein bands were visualized using enhanced chemiluminescence (ECL) and quantified using ImageJ software.

### 2.11 Quantitative real-time PCR (RT-qPCR) analysis

Total RNA was extracted from colon tissues using TRIzol reagent according to the manufacturer’s instructions. Briefly, approximately 100 mg of tissue was homogenized in TRIzol, followed by chloroform extraction and isopropanol precipitation. RNA pellets were washed with 75% ethanol, air-dried, and dissolved in RNase-free water. RNA concentration and purity were determined using a NanoDrop spectrophotometer, and samples were adjusted to a final concentration of 500 ng/μL.

cDNA was synthesized using the PrimeScript RT reagent kit according to the manufacturer’s protocol. Reverse transcription was performed at 37 °C for 15 min, followed by enzyme inactivation at 85 °C for 5 s. Specific amplification primers were designed for *Occludin*, *ZO−1*, *Claudin−4*, *TNF-α*, *IL-1β*, *IL-6*, *TLR4*, and *GAPDH* (internal reference gene) (Supplementary Table 1). RT-qPCR was performed using TB Green Premix Ex Taq II on a real-time PCR detection system. The amplification conditions were as follows: initial denaturation at 95 °C for 30 s, followed by 40 cycles of denaturation at 95 °C for 30 s and annealing/extension at 60 °C for 34 s. Relative mRNA expression levels were calculated using the ΔΔCt method with *GAPDH* as the internal reference gene.

### 2.12 Statistical analysis

All statistical analyses were conducted using IBM SPSS Statistics version 26.0 (IBM Corp., Armonk, NY, USA). Data are presented as mean ± standard deviation (SD). Repeated-measures analysis of variance (ANOVA) was used to assess within-group differences across multiple time points. One-way ANOVA was applied for comparisons among groups after testing for homogeneity of variance. Pairwise comparisons were conducted using Student’s *t*-test. A two-tailed P value < 0.05 was considered statistically significant. Graphs were generated using GraphPad Prism version 9.5 (GraphPad Software, San Diego, CA, USA).

## 3 Results

### 3.1 MWA treatment improves sleep and cognitive function in insomnia rats

To evaluate the therapeutic effects of MWA on insomnia, five experimental groups were established, and general physiological parameters, including body weight, sleep behavior, cognitive function, and anxiety-like behavior, were assessed before modeling, after model establishment, and after treatment.

**Fig. 2:**
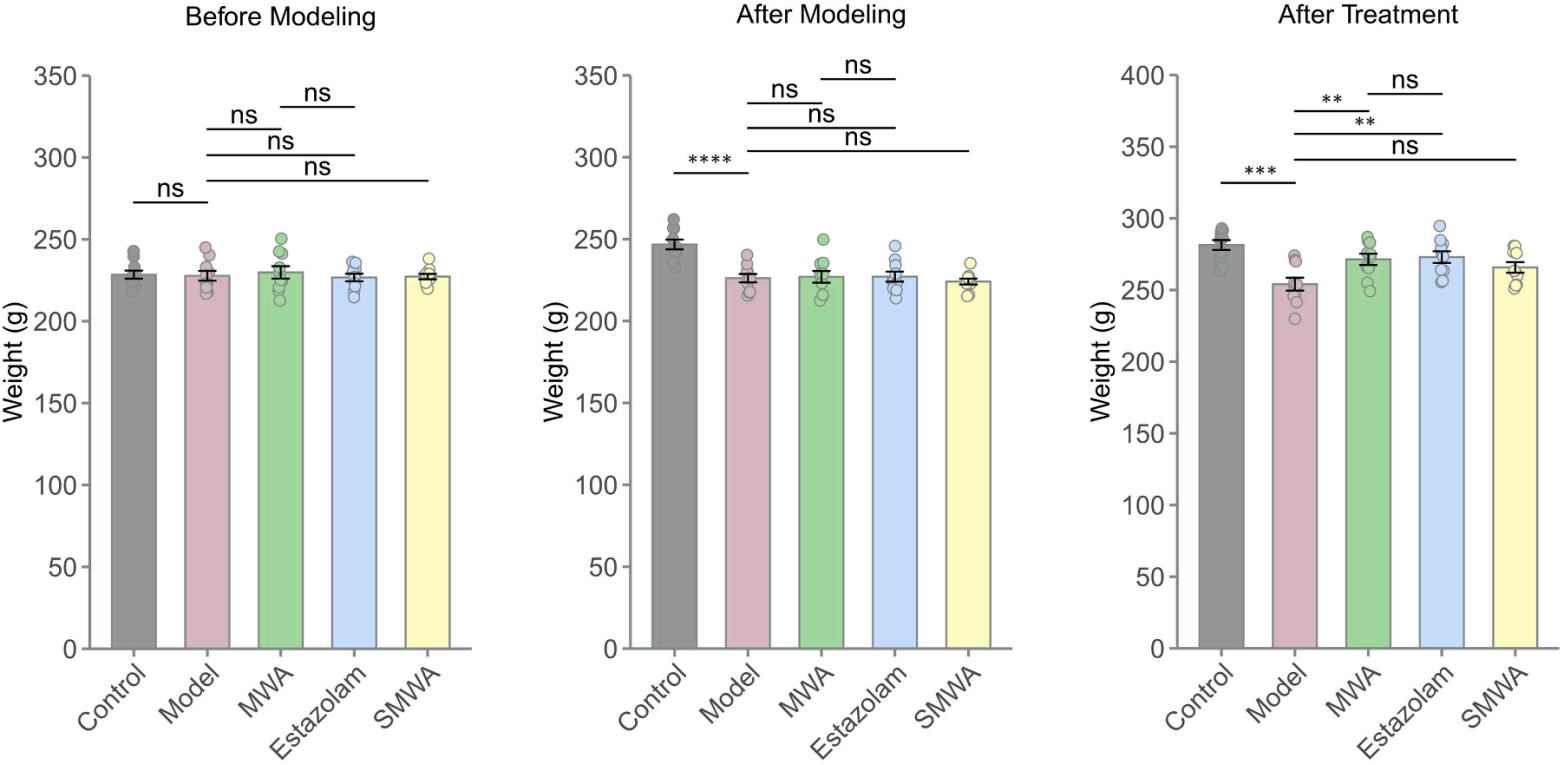
Body weight changes before modeling, after modeling, and after treatment among different groups. Data are presented as mean ± SD (n = 10 per group). Statistical significance was determined using one-way ANOVA followed by Tukey’s post hoc test. *P < 0.05, **P < 0.01, ***P < 0.001, ****P < 0.0001; ns, not significant.

No significant differences in body weight were observed among groups before model induction. After insomnia modeling, rats in the Model, MWA, Estazolam, and SMWA groups exhibited significantly lower body weight compared with the Control group, with no significant differences among the modeled groups. After 7 days of treatment, body weight in the MWA and Estazolam groups was significantly higher than that in the Model group, whereas the SMWA group showed no significant difference compared with the Model group (Fig. 2).

**Fig. 3:**
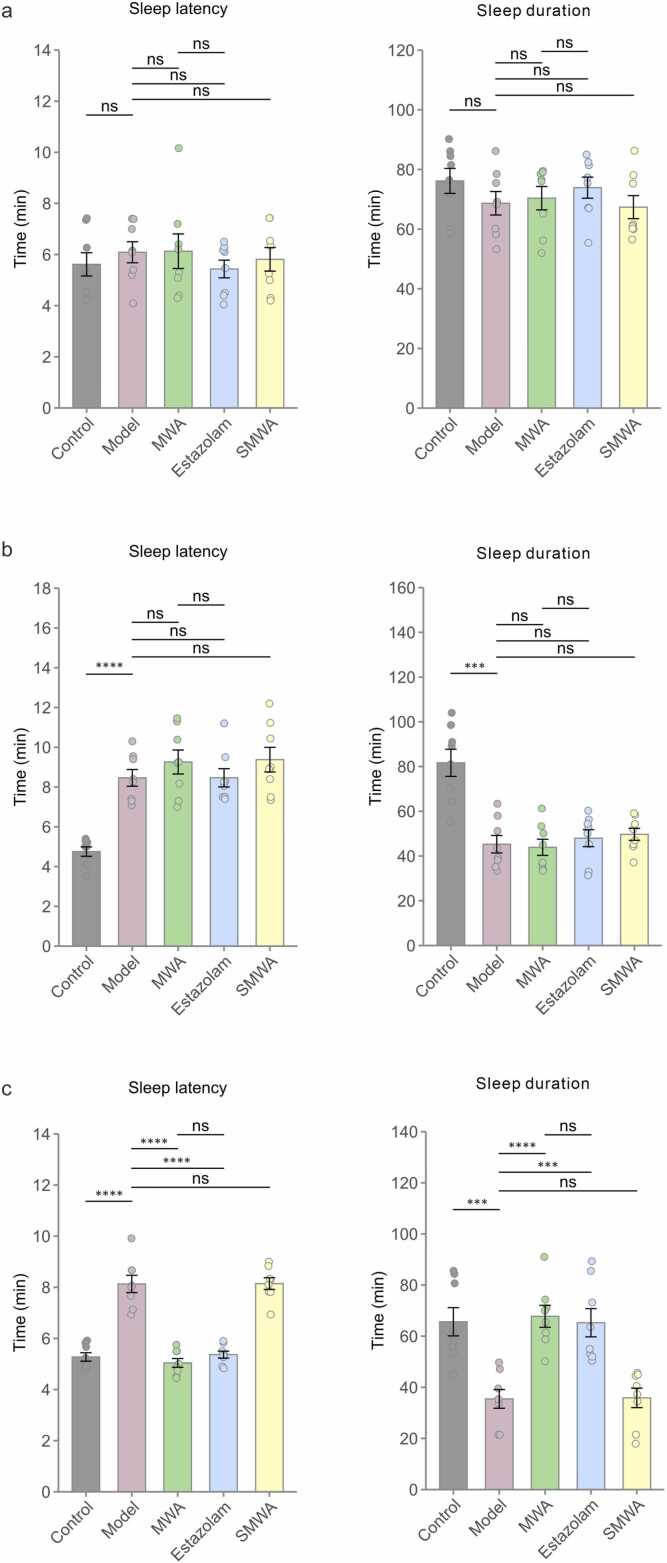
Pentobarbital sodium-induced sleep test before modeling, after modeling, and after treatment. Sleep latency and sleep duration were assessed using the pentobarbital sodium-induced sleep test in the Control, Model, MWA, Estazolam, and SMWA groups. (a) Sleep latency and sleep duration before model induction. (b) Sleep latency and sleep duration after insomnia model establishment. (c) Sleep latency and sleep duration after 7 days of treatment. Data are presented as mean ± SD (n = 10 per group). Statistical significance was determined using one-way ANOVA followed by Tukey’s post hoc test. ***P < 0.001, ****P < 0.0001; ns, not significant.

In the pentobarbital sodium-induced sleep test, no significant differences in sleep latency or sleep duration were observed among the groups before model induction (Fig. 3**a**). After model establishment, the Model, MWA, Estazolam, and SMWA groups showed significantly prolonged sleep latency and shortened sleep duration compared with the Control group, with no significant differences among the modeled groups (Fig. 3**b**). Following 7 days of treatment, both MWA and Estazolam groups showed significantly reduced sleep latency and increased sleep duration compared with the Model group, while no significant difference was observed between the SMWA and Model groups (Fig. 3**c**).

**Fig. 4:**
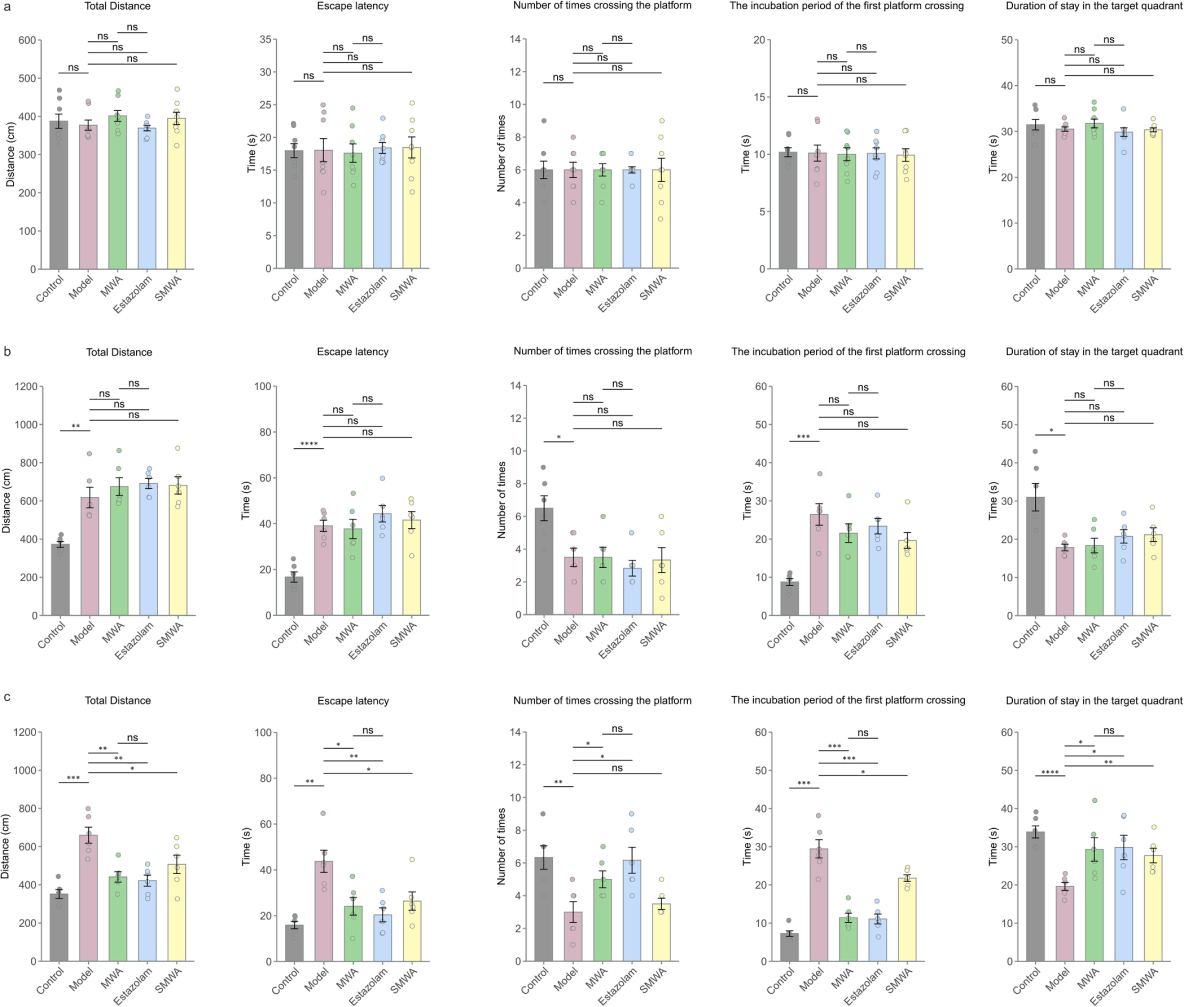
Morris water maze performance before modeling, after modeling, and after treatment. Spatial learning and memory were evaluated using the Morris water maze test in the Control, Model, MWA, Estazolam, and SMWA groups. (a) Behavioral performance before model induction. (b) Behavioral performance after insomnia model establishment. (c) Behavioral performance after 7 days of treatment. Parameters assessed included total swimming distance, escape latency, number of platform crossings, latency to the first platform crossing, and time spent in the target quadrant. Data are presented as mean ± SD (n = 10 per group). Statistical significance was determined using one-way ANOVA followed by Tukey’s post hoc test. *P < 0.05, **P < 0.01, ***P < 0.001, ****P < 0.0001; ns, not significant.

In the Morris water maze test, no significant differences were observed among the groups before model induction (Fig. 4**a**). Following insomnia model establishment, the Model, MWA, Estazolam, and SMWA groups exhibited significantly increased total swimming distance and escape latency, accompanied by reduced platform crossing frequency, decreased time spent in the target quadrant, and prolonged latency to the first platform crossing compared with the Control group, indicating successful induction of cognitive impairment. No significant differences were observed among the modeled groups at this stage (Fig. 4**b**). After 7 days of treatment, the MWA and Estazolam groups showed significantly reduced total swimming distance and escape latency compared with the Model group. In addition, both groups exhibited increased platform crossing frequency, prolonged time spent in the target quadrant, and reduced latency to the first platform crossing, indicating improved spatial learning and memory performance. In contrast, the SMWA group showed only limited improvement and remained significantly impaired compared with the MWA and Estazolam groups (Fig. 4**c**).

**Fig. 5:**
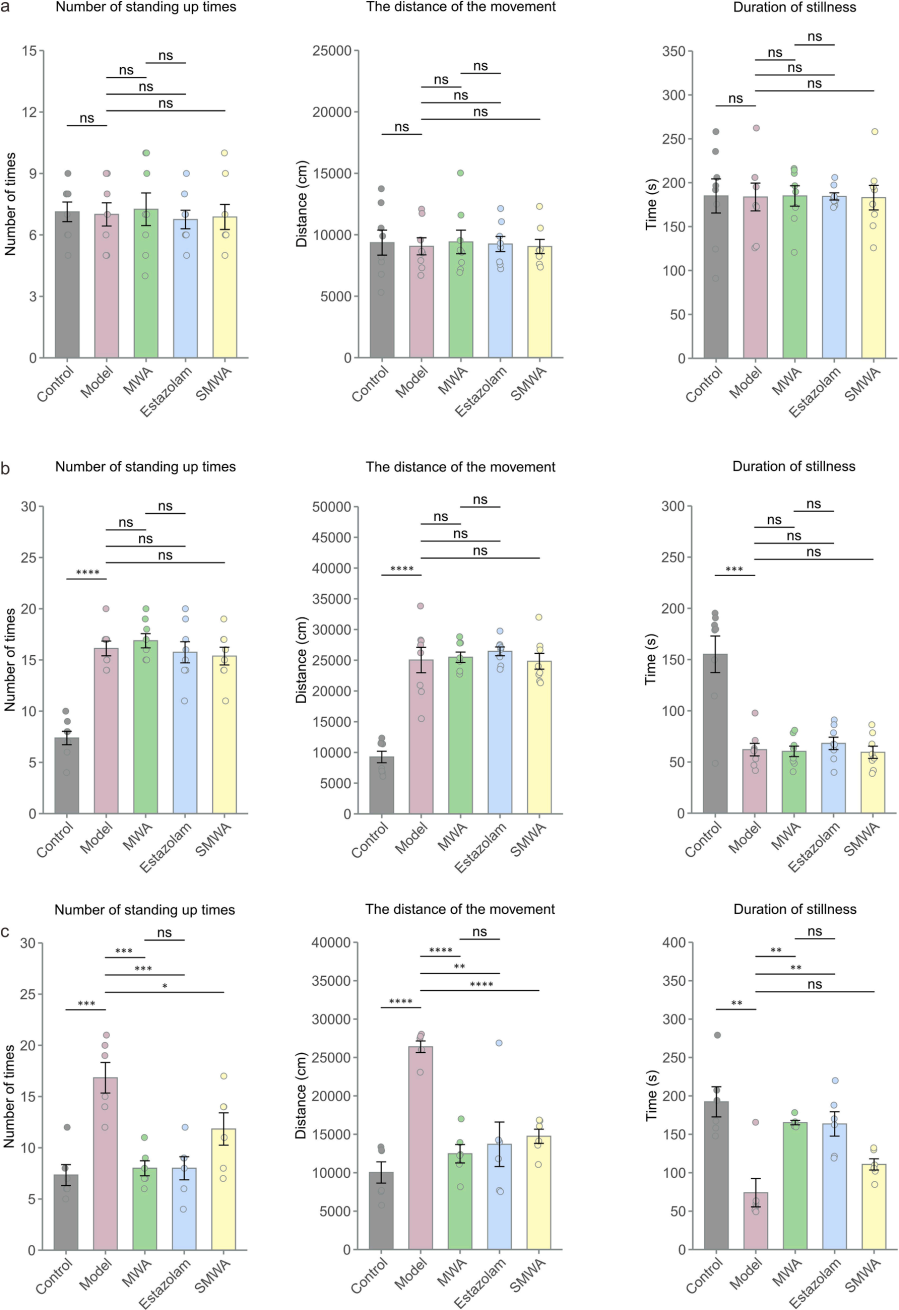
Open field test performance before modeling, after modeling, and after treatment. Behavioral activity was assessed using the open field test in the Control, Model, MWA, Estazolam, and SMWA groups. (a) Behavioral performance before model induction. (b) Behavioral performance after insomnia model establishment. (c) Behavioral performance after 7 days of treatment. Parameters assessed included rearing frequency, total locomotor distance, and immobility time. Data are presented as mean ± SD (n = 10 per group). Statistical significance was determined using one-way ANOVA followed by Tukey’s post hoc test. *P < 0.05, **P < 0.01, ***P < 0.001, ****P < 0.0001; ns, not significant.

In the open field test, no significant differences were observed among the groups before model induction in rearing frequency, total locomotor distance, or immobility time (Fig. 5**a**). Following insomnia model establishment, the Model, MWA, Estazolam, and SMWA groups exhibited significantly increased locomotor activity and rearing frequency, accompanied by reduced immobility time compared with the Control group (Fig. 5**b**). No significant differences were observed among the modeled groups at this stage. After 7 days of treatment, both the MWA and Estazolam groups significantly reduced locomotor distance and rearing frequency and increased immobility time compared with the Model group. The SMWA group showed reduced significantly locomotor distance but no significant differences in immobility time or rearing frequency compared with the Model group (Fig. 5**c**).

Together, the behavioral assessments demonstrated that MWA treatment significantly improved sleep behavior, cognitive performance, and anxiety-like behavior in insomnia rats.

### 3.2 MWA reduce histopathological damage in colon of insomnia rats

**Fig. 6:**
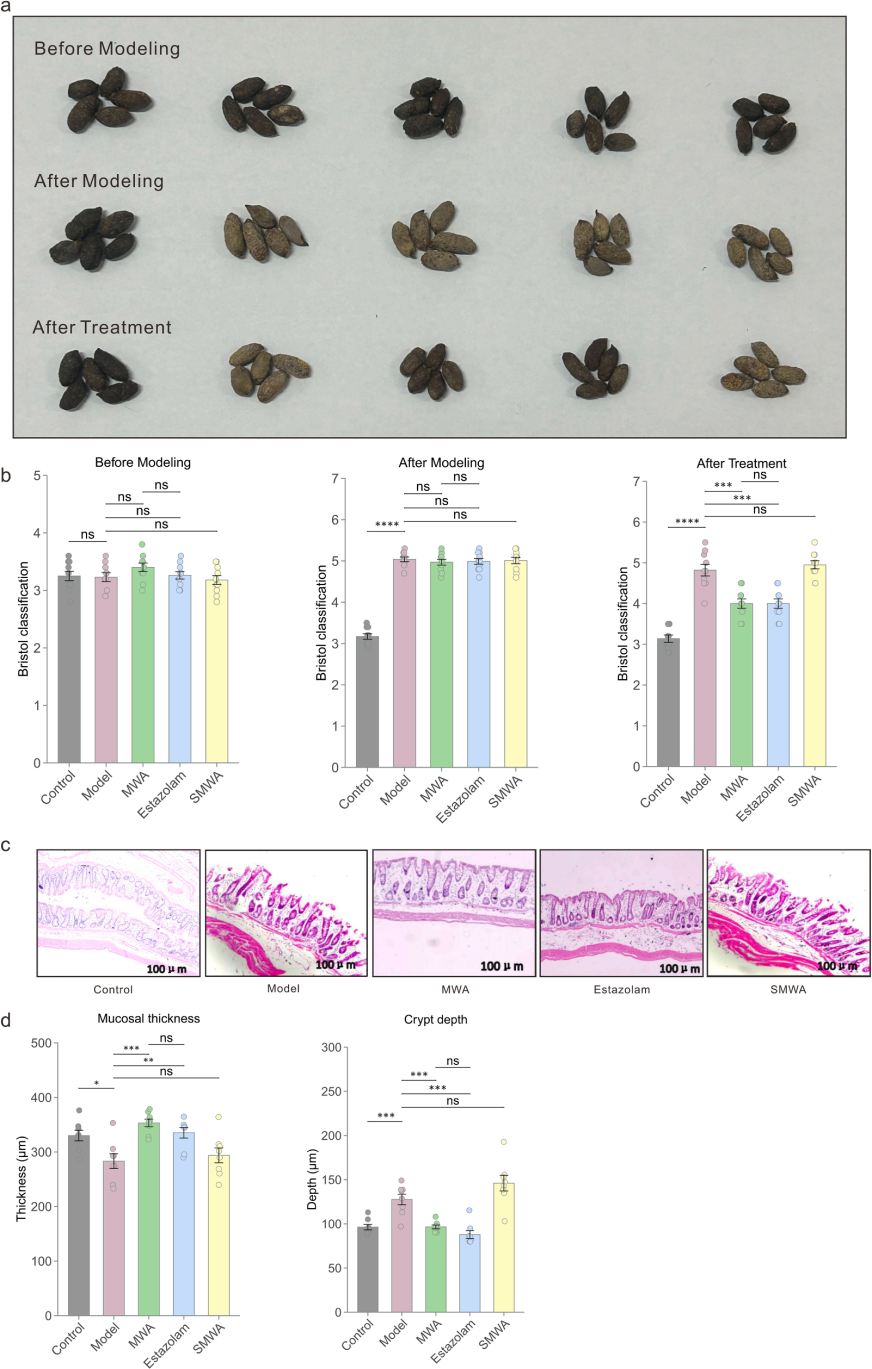
Effects of MWA on colon morphology and histopathological alterations in insomnia rats. (a) Representative fecal morphology at baseline, after model induction, and after treatment. (b) Bristol Stool Scale scores of fecal samples at the corresponding time points. Bristol Stool Scale scores ranged from 1 to 7, with higher scores indicating softer stool consistency. Scores 1–2 indicate hard stools/constipation-like features, scores 3–4 indicate normal stool consistency, scores 5–6 indicate soft or loose stools, and score 7 indicates watery stool. (c) Representative H&E staining of colonic tissues showing histopathological changes after treatment. Scale bar = 100 μm. (d) Quantitative analysis of mucosal thickness and crypt depth based on the histological images shown in panel. Data are presented as mean ± SD (n = 10 per group). *P < 0.05, **P < 0.01, ***P < 0.001, ****P < 0.0001; ns, not significant.

Representative fecal morphology was assessed and Bristol Stool Scale scores were recorded. No significant differences in Bristol scores were observed among groups before model induction. Following model establishment, insomnia-induced rats exhibited significantly increased Bristol scores, characterized by reduced fecal output, smaller and drier pellets, and lighter coloration compared with the Control group. After treatment, Bristol scores were significantly reduced in both the MWA and Estazolam groups compared with the Model group and returned to levels comparable to those of the Control group, whereas only minimal changes were observed in the SMWA group (Fig. 6**a-b**). Histopathological examination showed that colonic tissues in the Control group exhibited intact mucosal architecture without obvious inflammatory cell infiltration. In contrast, the Model group showed obvious mucosal disruption, reduced mucosal thickness, increased crypt depth, and inflammatory cell infiltration. After treatment, colonic histological architecture was substantially restored in both the MWA and Estazolam groups, accompanied by increased mucosal thickness and reduced crypt depth. In contrast, the SMWA group showed similar histopathological features to the Model group (Fig. 6**c-d**).

### 3.3 MWA improves colonic barrier function in insomnia rats

**Fig. 7:**
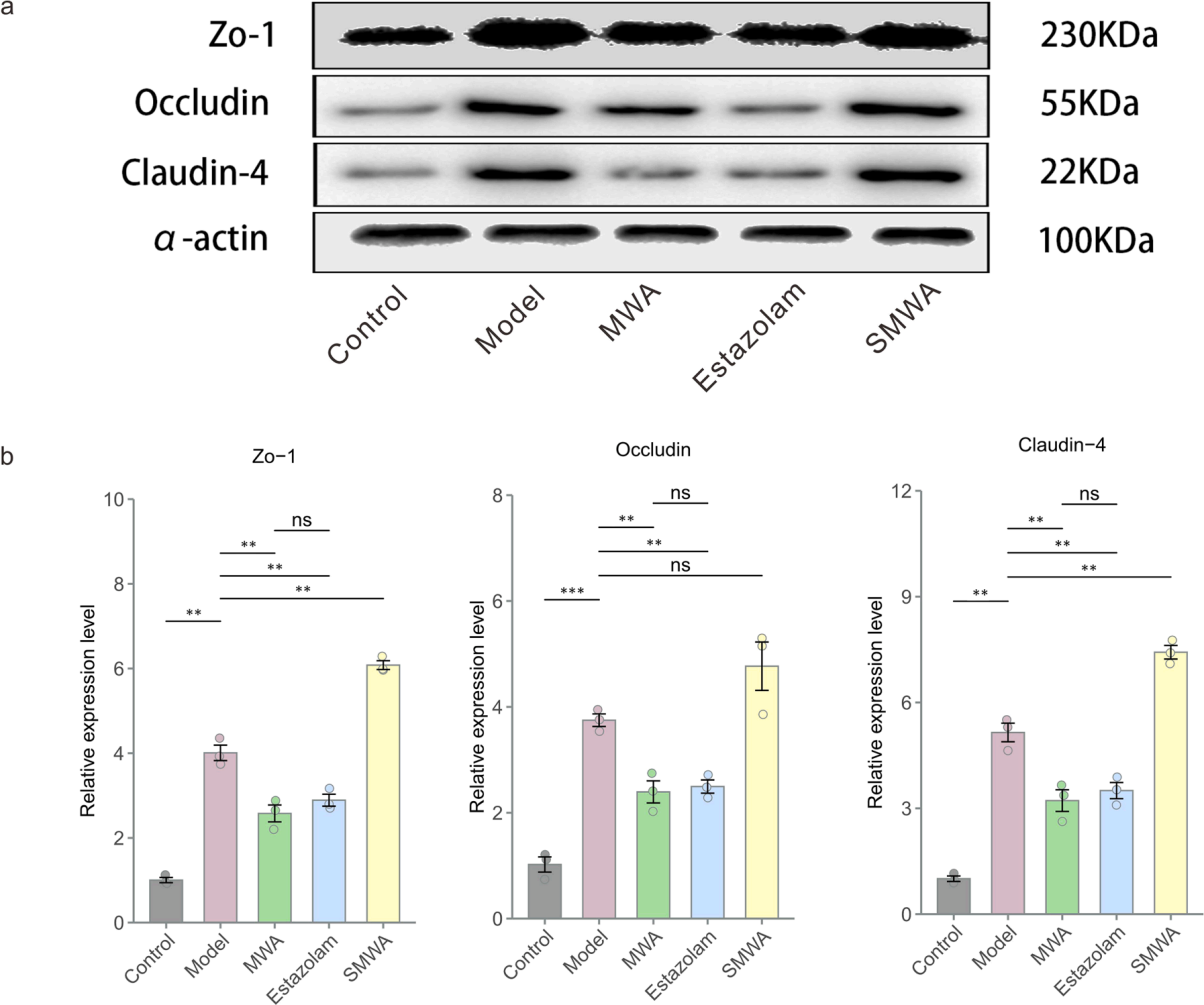
MWA improves intestinal barrier-related protein expression in insomnia rats. (a) Representative Western blot images showing the protein expression levels of ZO−1, Occludin, and Claudin−4 in colonic tissues from different groups. α-actin was used as the loading control. (b) RT-qPCR of protein expression levels normalized to α-actin. Data are presented as mean ± SD (n = 6 per group). Statistical analysis was performed using one-way ANOVA followed by Tukey’s post hoc test. *P < 0.05, **P < 0.01, ***P < 0.001; ns, not significant.

To assess the effects of MWA treatment on colonic permeability, the expression levels of tight junction proteins ZO−1, Occludin, and Claudin−4 were evaluated using Western blot and RT-qPCR. Western blot analysis showed that the protein levels of ZO−1, Occludin, and Claudin−4 were significantly increased in the Model group compared with the Control group. After treatment, both the MWA and Estazolam groups significantly reduced protein levels compared with the Model group. The SMWA group showed no significant differences in Occludin and Claudin−4 expression compared with the Model group, while ZO−1 expression was slightly decreased. No significant differences were observed between the MWA and Estazolam groups (Fig. 7**a**).

Consistent with the protein expression results, RT-qPCR analysis showed that the mRNA expression levels of *ZO−1*, *Occludin*, and *Claudin−4* were significantly increased in the Model group compared with the Control group. Both the MWA and Estazolam groups showed significantly reduced mRNA expression levels compared with the Model group. The SMWA group showed no significant change in protein expressions compared with the Model group. No significant differences were observed between the MWA and Estazolam groups (Fig. 7**b**). These results suggest that MWA ameliorates intestinal barrier dysfunction in insomnia rats.

### 3.4 MWA improves colonic inflammatory responses in insomnia rats

**Fig. 8:**
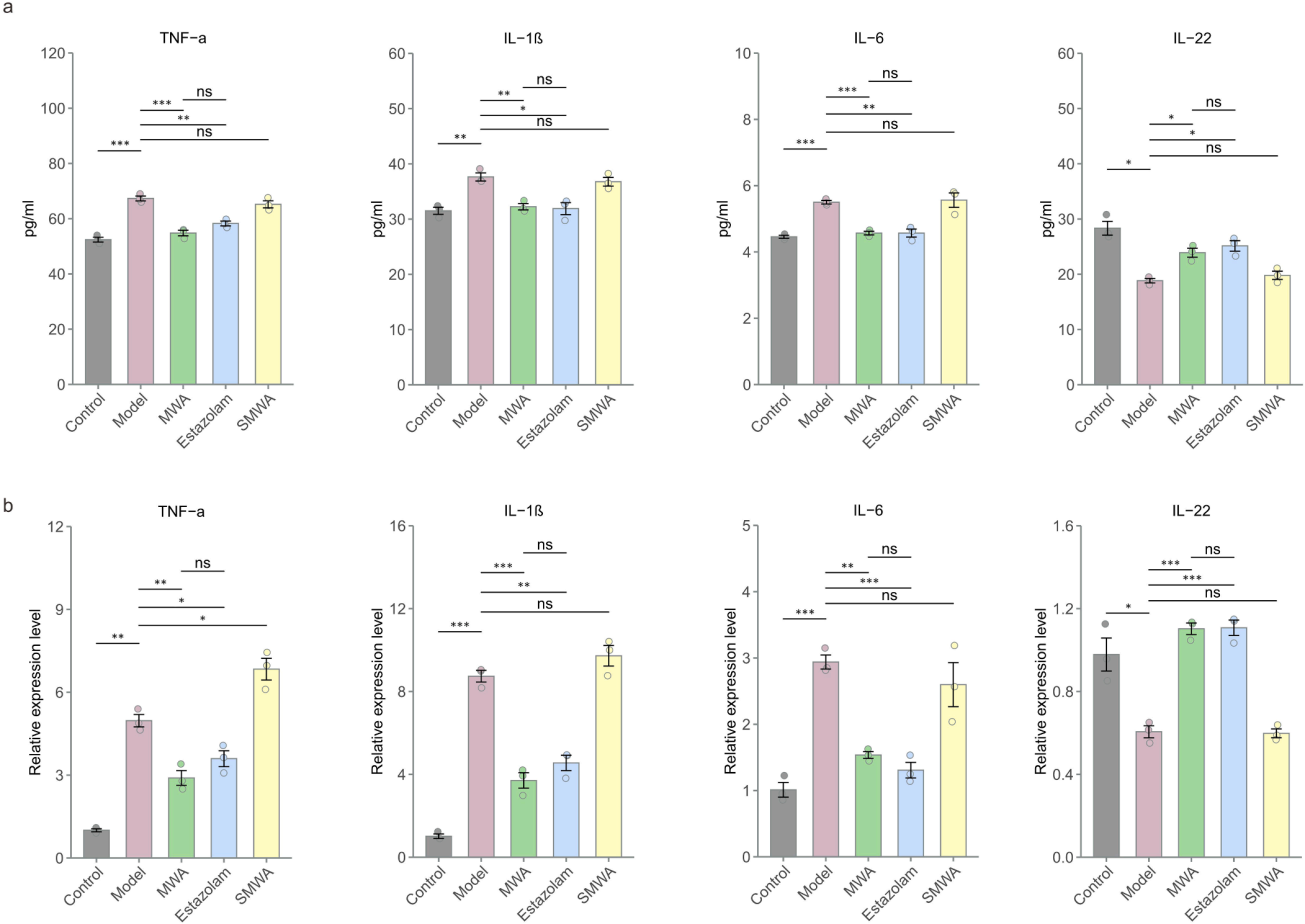
MWA regulates inflammatory cytokine expression in the colonic tissues of insomnia rats. (a) ELISA analysis of TNF-α, IL-1β, IL-6, and IL-22 levels in colonic tissues. (b) Relative mRNA expression levels of TNF-α, IL-1β, IL-6, and IL-22 determined by RT-qPCR. Data are presented as mean ± SD (n = 6 per group). Statistical analysis was performed using one-way ANOVA followed by Tukey’s post hoc test. *P < 0.05, **P < 0.01, ***P < 0.001; ns, not significant.

To evaluate the effects of MWA treatment on colonic immune responses, the protein levels and mRNA expression of inflammatory cytokines TNF-α, IL-1β, IL-6, and IL-22 were assessed using ELISA and RT-qPCR. ELISA results showed that that the Model group exhibited significantly increased levels of TNF-α, IL-1β, and IL-6 and decreased IL-22 levels compared with the Control group. Both MWA and Estazolam groups significantly reduced TNF-α, IL-1β, and IL-6 levels and increased IL-22 levels compared with the Model group, whereas the SMWA group showed no significant differences. No significant differences were observed between the MWA and Estazolam groups (Fig. 8**a**).

Consistent with the ELISA results, RT-qPCR analysis showed that mRNA expression levels of TNF-α, IL-1β, and IL-6 were significantly increased, whereas IL-22 expression was significantly decreased in the Model group compared with the Control group. Both MWA and Estazolam groups significantly reversed these changes, while the SMWA group showed no significant differences compared with the Model group. No significant differences were observed between the MWA and Estazolam groups (Fig. 8**b**). These results suggest that MWA improves colonic inflammatory responses in insomnia rats.

### 3.5 MWA regulates the TLR4/MyD88/NF-κB signaling pathway in insomnia rats

**Fig. 9:**
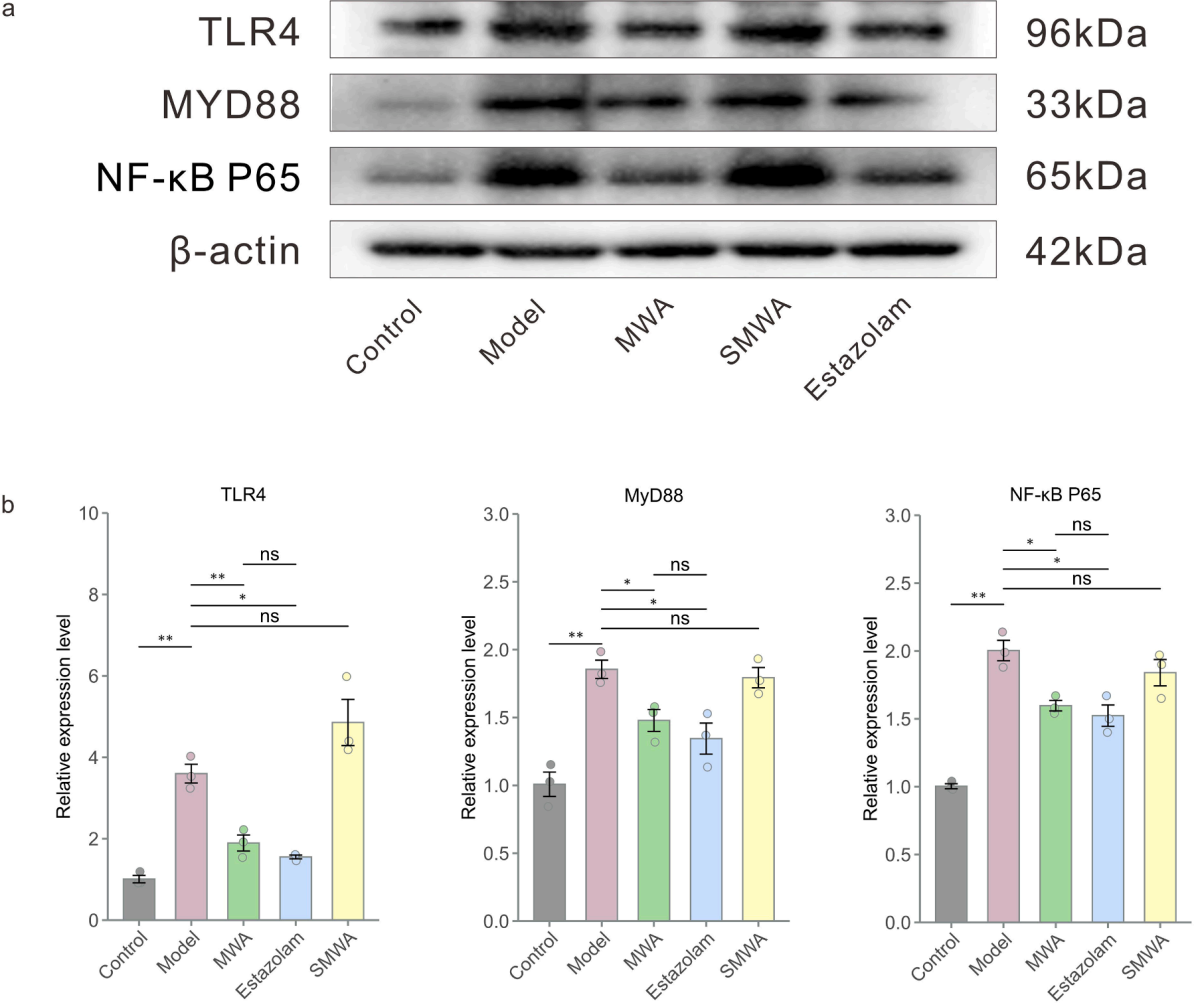
MWA regulates the TLR4/MyD88/NF-κB signaling pathway in colonic tissues of insomnia rats. (a) Protein expression levels of TLR4, MyD88, and NF-κB p65 in colonic tissues by Western blotting. β-actin was used as the loading control. (b) RT-qPCR of relative protein expression levels normalized to β-actin. Data are presented as mean ± SD (n = 6 per group). Statistical analysis was performed using one-way ANOVA followed by Tukey’s post hoc test. *P < 0.05, **P < 0.01; ns, not significant.

To further investigate the molecular mechanisms underlying the protective effects of MWA treatment, the expression of key components of the TLR4/MyD88/NF-κB signaling pathway was assessed by Western blotting and RT-qPCR. Western blot analysis showed that protein levels of TLR4, MyD88, and NF-κB p65 were significantly increased in the Model group compared with the Control group. Following treatment, both the MWA and Estazolam treatment groups showed significantly reduced protein levels compared with the Model group, whereas the SMWA group showed no significant differences (Fig. 9**a**).

Consistent with the protein results, RT-qPCR analysis showed that the mRNA expression levels of TLR4, MyD88, and NF-κB p65 were significantly increased in the Model group compared with the Control group. Both MWA and Estazolam treatment groups significantly reduced the genes expression levels compared with the Model group, whereas the SMWA group showed no significant changes. No significant differences were observed between the MWA and Estazolam groups (Fig. 9**b**). These findings indicate that MWA modulates TLR4/MyD88/NF-κB signaling in insomnia rats.

## 4 Discussion

Insomnia is increasingly recognized as a systemic disorder involving neuroimmune and gut-brain axis dysregulation (Cheng et al., 2025). In the present study, we demonstrated that MWA treatment significantly improved sleep behavior, intestinal morphology, barrier integrity, inflammatory responses, and TLR4/MyD88/NF-κB signaling activation in insomnia rats. These findings suggest that Mongolian warm acupuncture exerts multi-level regulatory effects on intestinal barrier and immune homeostasis under insomnia conditions.

In our study, insomnia modeling induced by chronic stress combined with PCPA resulted in typical insomnia-like phenotypes, including reduced body weight, prolonged sleep latency, shortened sleep duration, impaired spatial learning and memory, and increased anxiety-like behavior. These findings are consistent with previous reports showing that chronic stress and serotonin depletion can induce sleep disturbance and cognitive dysfunction in rodent models (Ren et al., 2020, Huitrón-Reséndiz et al., 1997). After treatment, MWA markedly improved sleep latency and sleep duration, accompanied by improvements in spatial learning ability and anxiety-like behaviors (Fig. 2, Fig. 3, Fig. 4, Fig. 5**)**. Notably, the therapeutic effects of MWA were comparable to those of estazolam in several behavioral parameters, suggesting that MWA may represent a potential alternative or complementary therapeutic strategy for insomnia management. In addition, MWA showed greater improvement in memory retention-related parameters compared with SMWA treatment, indicating that the therapeutic effects were not solely due to nonspecific stimulation or handling-related stress reduction. This supports the specificity of MWA intervention in modulating central nervous system function under insomnia conditions.

Sleep deprivation and chronic stress have been reported to disrupt intestinal barrier function and promote low-grade systemic inflammation (Veler, 2023). In our study, insomnia model rats showed obvious intestinal structural damage accompanied by abnormal regulation of tight junction proteins, including ZO−1, Occludin, and Claudin−4 (Figs. 6 and 7). Tight junction proteins are essential for maintaining intestinal epithelial barrier integrity and preventing excessive luminal antigen translocation (Turner, 2009). Dysregulation of tight junction proteins under pathological conditions may represent a compensatory response to barrier injury or inflammatory stimulation (Chelakkot et al., 2018). Our findings showed that MWA restored tight junction protein expression toward normal physiological levels, suggesting its protective role in maintaining intestinal barrier homeostasis.

Inflammatory cytokines are critical mediators linking intestinal barrier dysfunction and systemic immune activation (Peterson and Artis, 2014). We observed significantly increased pro-inflammatory cytokines (TNF-α, IL-1β, and IL-6) and decreased IL-22 levels in insomnia model rats (Fig. 8). IL-22 is known to promote epithelial regeneration and mucosal barrier repair (Patnaude et al., 2021). MWA treatment significantly reversed these inflammatory alterations, indicating that it may attenuate intestinal inflammation while promoting mucosal immune protection.

The TLR4/MyD88/NF-κB signaling pathway plays a central role in innate immune activation and inflammatory amplification (Yu et al., 2020). Activation of TLR4 signaling can trigger downstream MyD88-dependent NF-κB activation, leading to transcription of multiple pro-inflammatory mediators (Zhao et al., 2021). In the present study, insomnia modeling significantly activated TLR4/MyD88/NF-κB signaling, whereas MWA treatment markedly inhibited this pathway (Fig. 9), suggesting that the anti-inflammatory and barrier-protective effects of MWA may be mediated, at least in part, through suppression of TLR4/MyD88/NF-κB signaling activation.

In conclusion, our findings indicate that MWA treatment may alleviate insomnia-associated intestinal barrier dysfunction and inflammatory responses through modulation of gut immune signaling pathways. This provides mechanistic evidence supporting the therapeutic potential of MWA in insomnia treatment.

## Ethics approval and consent to participate

All methods and materials were in compliance with relevant institutional, national, and international guidelines and legislation.

## Authors' contributions

H.Y. had the idea for and designed the study. A. supervised the study. G.,Z.B. and Z.W. did the statistical analysis. All authors contributed to acquisition, analysis, or interpretation of data. G. wrote the draft report. All authors revised the report and approved the final version before submission.

## Authors agreement

The authors declare that there is no conflict of commercial interest related to this paper.

## Consent for publication

Not applicable.

## Funding

This project was supported by The National Natural Science Foundation of China (82460987), Natural Science Foundation of Inner Mongolia Autonomous Region of China (2023QN08065,2025QN08018), ‌Baotou Medical College Scientific Research Fund Projects (BYJJ-BSJJ 202406,BYJJ-GCC202508).

## Conflict of Interest

The authors declare that there is no conflict of commercial interest related to this paper.

## Data Availability

All data and materials mentioned in this article are available.
