# Supplementary material for: Mongolian warm acupuncture improves colonic barrier function and immune responses via the TLR4/MyD88/NF-κB pathway in insomnia rats
Source: IBRO Neurosci Rep. 2026 Jul 25;21:401–12. doi: 10.1016/j.ibneur.2026.07.014 (PMC13452145; doi:10.1016/j.ibneur.2026.07.014)
Supplement: Supplementary file 1 [file mmc1.docx]

**Supplementary Table 1. Primer sequences used for RT-qPCR**

| Gene | Forward (5’ – 3’) | Reverse (5’ - 3’) |
| --- | --- | --- |
| *GAPDH* | ACAGCAACAGGGTGGTGGAC | TTTGAGGGTGCAGCGAACTT |
| *Zo-1* | GCAAAGAGATGAGCGGGCTACC | GGGAGCGAACTGAATGGTCTGATG |
| *Claudin 4* | TCGGCTGGGCGGCTTCTG | TTACACATAGTTGCTGGCGGGAAC |
| *Occludin* | CAACGGCAAAGTGAATGGCAAGAG | TCATCCACGGACAAGGTCAGAGG |
| *TLR4* | GGTTGGCACTCTCACTTCCTCTTG | GTAAATGGTGGCAGGGCAGAGTC |
| *TNF-α* | AAAGGACACCATGAGCACGGAAAG | CGCCACGAGCAGGAATGAGAAG |
| *IL-6* | ACTTCCAGCCAGTTGCCTTCTTG | TGGTCTGTTGTGGGTGGTATCCTC |
| *IL-1β* | TCGCAGCAGCACATCAACAAGAG | AGGTCCACGGGAAAGACACAGG |
